# Sprengel Deformity in Biological Sisters

**DOI:** 10.5435/JAAOSGlobal-D-19-00120

**Published:** 2020-04-02

**Authors:** Carlos Pargas, Adolfredo Santana, Wojciech L. Czoch, Kenneth J. Rogers, William G. Mackenzie

**Affiliations:** From the Department of Orthopaedic Surgery, Nemours/Alfred I. duPont Hospital for Children, Wilmington, DE.

## Abstract

Sprengel deformity is the main congenital shoulder deformity seen in children. First described by Otto Sprengel in 1891, it consists of a high-riding, dysplastic, and malrotated scapula leading to functional and cosmetic impairments. It has never been reported in siblings.

**Case Presentation:**

Two sisters, 8 and 9 years old, presented for an evaluation of atraumatic limitation in the shoulder range of motion and neck webbing with an unknown family history. Physical examination revealed a small high-riding scapula, webbed neck, and painless limitation in shoulder abduction (<70°) and flexion (<80°). The 9-year-old sibling had a bilateral shoulder involvement, and the younger had unilateral. Imaging revealed bony and fibrous omovertebral connections between the dysplastic scapulas and cervical spine along with Klippel-Feil deformities. Both sisters underwent scapula repositioning via a modified Woodward procedure. The omovertebral connection was resected followed by scapula derotation and inferior migration. Both had a dramatic improvement in cosmesis and near-complete restoration of shoulder function at follow-up.

**Conclusions:**

Although uncommon, Sprengel deformity results in notable derangement of shoulder function. If untreated, children experience difficulty with most overhead activities and often have cosmetic reports. Although no previous genetic link has been identified, its presence in biological sisters suggests that more research is needed.

Sprengel deformity is a rare shoulder girdle anomaly; however, it is the most common congenital shoulder deformity seen in children. Michael Eulenberg initially described it in 1863 in the German journal Chirurgie. He presented a single case of an undescended scapula, described its anatomy, and suspected a traumatic etiology.^1,2^ Approximately 30 years later, Otto Gerhard Karl Sprengel expanded on the publication of Eulenberg by reporting four clinical cases of dysplastic and undescended scapula. Unlike Eulenberg, Sprengel was the first to propose that the anomaly had a congenital origin and described its associated pathology.^3^

Today, the condition is widely accepted to be of congenital origin where there is a failure of formation and subsequent descent leading to a high-riding, dysplastic, and malrotated scapula. The scapula is rotated anteriorly/forward, the convex side translated medially, which articulates with the inferior and lateral aspect of the omovertebral bone. The glenoid surface is orientated anteroinferiorly. Anterior-posterior radiographic views show the normal scapula to have a triangular shape, whereas with Sprengel deformity the shape is rhomboid. Its cause remains largely unknown, but interruption of embryonic blood supply because of vascular lesions arising from the subclavian artery has been proposed.^3^ In addition, its association with Klippel-Feil syndrome, scoliosis, cardiopulmonary malformation (occurrence of 4% to 14%), and genitourinary malformation (occurrence of 35%) has been well described.^4,5^ Left untreated, Sprengel deformity will often lead to functional and cosmetic impairments such as restricted shoulder motion and pronounced ipsilateral neck webbing.

In addition to the triad of pathoanatomy previously described, approximately 20% to 50% of patients with Sprengel deformity will have an abnormal bony or fibrous bridge between the undescended scapula and the cervicothoracic spine.^6^ Termed the omovertebral bone, its origin remains unclear but it serves to further restrict scapula motion and impair shoulder function.

The Cavendish classification, initially proposed in 1972, is based on the deformity and does not consider the function. Grade 1 is described as a very mild deformity that is not noticeable when the patient is dressed. Grade 2 is described as a mild deformity that is visible as a lump in the web of the neck when the patient is dressed. Grade 3 is a moderate deformity described as an easily visible deformity with the shoulder joint elevated 2 to 5 cm. Grade 4 is a severe deformity with a shoulder joint elevation greater than 5 cm or evidence of the superior angle of the scapula near the occiput with or without webbing.^4,7,8^

This classification remains the most widely used on the presence of clinical deformity.^9^ Because this classification does not take into account functional impairment, it serves a limited role for treatment guidance and outcome prediction. In addition, the Rigault classification is sometimes used to describe the radiographic position of the scapula.^6^

The treatment of Sprengel deformity is largely based on the age of the child and the degree of functional impairment. At times, severe cosmetic deformity will also warrant orthopaedic intervention. Typically, the condition is observed in infants and older children with mild loss of shoulder function. In children with moderate to severe functional impairment, surgical intervention and correction is often recommended.^9,10^

Several different surgical techniques are described in the literature. Today, the most popular approaches are the Green and Woodward procedures.^2,11^ Each method focuses on the scapula migration closer to its anatomic position. Relocation of the scapula with resection of anomalous tethers has been shown to provide notable improvement in function, appearance, and overall satisfaction.^10,12^

Although rare, the true incidence of Sprengel deformity remains unknown. The literature largely consists of independent case reports and expert opinion. In this study, we describe two sisters who presented with the same variant of Sprengel deformity. One experienced bilateral involvement and the younger sibling had unilateral involvement. Both sisters had well-established bony omovertebral connections. Owing to notable functional impairment, both patients underwent surgical correction via the Woodward procedure. To our knowledge, there are no previous reports of Sprengel deformity identified in biological siblings. Compliance of institutional regulations was followed for report consent.

## Case 1

This is a 9-year-old girl who presented to our clinic for evaluation of bilateral limitation in shoulder range of motion and function. The child's medical history was normal, and there were no reports of any injury or trauma. Her mother's pregnancy was normal, and she met all developmental milestones. On presentation, she did not report any pain and her greatest report was the inability to brush her hair.

**Figure 1:**
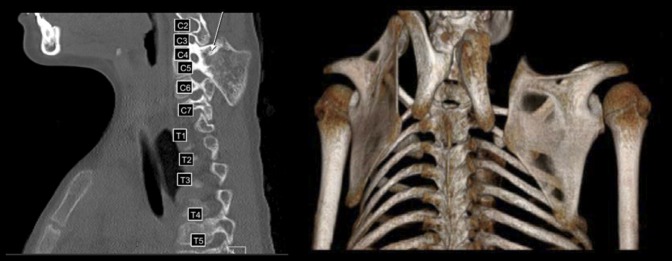
Photographs of two-dimensional CT sagittal view and three-dimensional CT view showing bilateral Sprengel deformities. Bilateral omovertebral bones, which proximally had an osseous connection to the scapula on the left side and fibrous on the right. Omevertebral bones are fused with posterior elements of the cervical spine.

Physical examination was consistent with pronounced bilateral neck webbing. Shoulder abduction was limited to 70° with 80° of forward flexion. Her scapulas were noted to be hypoplastic and symmetrically high riding. Plain radiographs and subsequent three-dimensional CT confirmed the presence of bilateral Sprengel deformity. Well-defined bony and fibrous connections between the scapula and the cervical spine were noted. The omovertebral bone was fused to the posterior elements involving the second to the sixth cervical (C2-C6) levels. In addition, the left omovertebral bone had an osseous connection proximally to the scapula and the right side, a fibrous connection (Figure 1). Multiple cervical vertebral segmentation anomalies were identified with involvement of the upper thoracic spine to the level of the second thoracic vertebrae as well. In addition, she was noted to have a Klippel-Feil deformity. Owing to her level of functional impairment, surgical intervention was indicated via the modified Woodward procedure.

## Case 2

This is an 8-year-old girl sibling of case 1 who was diagnosed with Klippel-Feil syndrome and Sprengel deformity at 4 months of age. The prenatal and neonatal period were normal, and she met all developmental milestones. Surgical history was relevant for cardiac issues.

**Figure 2:**
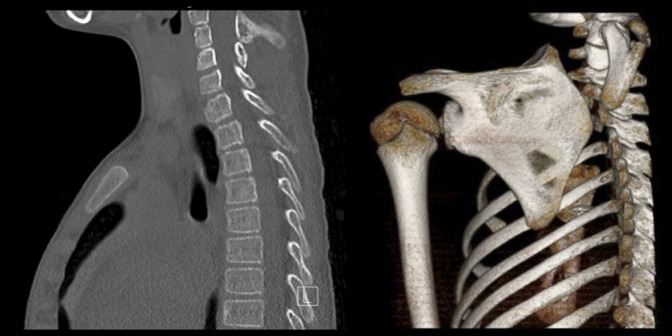
Photographs showing the left-sided Sprengel deformity with omovertebral bone and Klippel-Feil.

During follow-up, persistent webbing of the left side of the neck, asymmetrical shoulders (left shoulder higher than the right), and decreased range of motion (abduction and forward flexion) of the left shoulder (90°) compared with the opposite side (160°) was noticed in her physical examination. The left scapula was noted to be hypoplastic and high riding. At the C5 level, imaging studies showed a midline vertebral body cleft, thickening of the left lamina, nonfusion of the posterior elements, and the lateral aspect articulating with the left scapula, which was abnormally elevated (Figure 2). In addition, a 23° right-sided thoracic curve is also present. Owing to her functional impairment, surgical intervention was indicated.

## Surgical and Postoperative Time

The modified Woodward procedure was performed via the standard posterior midline incision. Intraoperatively, fluoroscopic PA view of shoulder, neck, and chest was used to identify the current and desired anatomic positions of the scapula. The superior aspect of the scapula is at the level of the second rib, and the inferior at the level of the sixth to seventh rib. In Sprengel deformity, we attempted to achieve a normal position of the scapula. However, appearance and satisfactory shoulder motion are the main goals. The omovertebral bones were then carefully identified and resected along with their spinal and scapula attachments. Next, the true medial borders of the scapula were identified. To assist with repositioning, the vertebral attachments of the trapezius, rhomboids, and levator scapulae were identified and taken down. Moderate fatty atrophy of the rhomboid muscle bellies was noted. The scapula was then derotated and globally repositioned into its anatomic location on the thoracic cavity. Small pointed reduction clamps were used for provisional anchoring to the spinous processes. Image intensification was used for confirmation. Using nonabsorbable suture, the detached muscle origins were then fixed to the more inferior vertebral levels. As a modification to the original Woodward procedure, we resected the superomedial angle (partial supraspinatus fossa resection) of the scapula to achieve an improvement appearance and shoulder functional results.^11^ In addition, intraoperative neuromonitoring was performed to identify any abnormal stress on the neural structures. In case 1, the signal amplitude of the left deltoid muscle decreased because the scapula was brought inferiorly and the arm was abducted. To reduce tension, the arm was adducted to a neutral position and the signal returned to normal.

Both patients remained in the hospital for 2 days postoperatively for pain control. They were placed in upper extremity shoulder immobilizers (one patient required bilateral immobilization) with daily elbow, wrist, and finger exercises to improve ranges of joint movement. The shoulder immobilizers were discontinued on postoperative week 3, and active and passive shoulder range of motion was begun with physical therapy after the incisions had healed adequately. Pendulum movements, flexion-extension, and abduction-adduction of the shoulder were incorporated progressively.

At 2 months postoperatively, both patients were noted to have near-full-shoulder abduction with total improvement in the arc of motion of nearly 120° and forward flexion was fully restored. They were able to easily reach their hands to the posterior occiput. They exhibited 4.5/5 muscle strength and reported no pain. There was moderate improvement in the bilateral neck webbing. Both patients and parents were quite pleased with the ultimate outcome.

## Discussion

Sprengel deformity is a rare congenital shoulder girdle anomaly, described initially by Eulenberg in 1863. In 1891, Otto Sprengel suggested an etiology to explain the most common characteristic of this deformity: scapula malposition (hypoplastic), periscapular muscle atrophy, and limited shoulder movements.^13^ The normal anatomic location of the scapula is in the posterior wall of the chest, between the second and the seventh and the eighth thoracic vertebrae. It is formed around the fifth week of gestation.^7^ In Sprengel deformity (hypoplastic rotated scapula), we attempted to achieve a normal position of the scapula. However, appearance and satisfactory shoulder motion are the main goals.

In the classic literature on Sprengel deformity, the scapula is hypoplastic and the ratio of horizontal-to-vertical diameter is increased. The muscles controlling scapula motion (trapezius, levator scapulae, and the rhomboids) are often contracted, atrophic, or infiltrated with fibrofatty tissue. In 16% to 55% of cases, there is an associated omovertebral tether that may be fibrous, chondral, or osseous and connects the scapula to the cervical spine. Usually, this connection extends from the superior medial angle of the scapula to the lower cervical spine, around C5 or C6.^6^

Traditionally, Sprengel deformity has been managed by omovertebral bar excision and muscle transplantation procedures guided by age and Cavendish grade; although this classification does not take into account the functional impairment, it remains the most used. For mild deformities, nonsurgical options, such as physical therapy and stretching, with the idea of preventing torticollis and keeping the range of motion are recommended. The moderate and severe deformities, classified as Cavendish grades 3 and 4, are candidates for surgical intervention. Many surgical procedures for Sprengel deformity correction have been discussed in the literature, but the hallmark techniques involve the resection of the omovertebral bone, if present, with caudal relocation of the scapula.^3,4,14^

One of the main characteristics of Sprengel deformity is the restriction of scapulothoracic motion, which in turn restricts shoulder abduction leading to an impairment that causes the cephalic position of the deformed scapula.^8^

Postoperative CT would certainly define the accuracy of the procedure; however, there would be radiation exposure without the ability to make a change. If there is a question of adequate correction during surgery, intraoperative evaluation seems more appropriate.

## Conclusion

Although uncommon, Sprengel deformity results in a notable derangement of shoulder function. If left untreated, children experience difficulty with most overhead activities and often have cosmetic reports. Although no previous genetic link has been identified, its presence in the biological sisters suggests that more research is needed in this field.
